# Parenting Stress and Stressful Life Events Among Caregivers of Toddler Siblings of Autistic and Non-Autistic Children

**DOI:** 10.1002/aur.70217

**Published:** 2026-03-12

**Authors:** Jennifer E. Magnuson, Lucy S. King, Jacob I. Feldman, S. Madison Clark, Grace Pulliam, Kacie Dunham-Carr, Alexandra Golden, Bahar Keçeli- Kaysılı, Kathryn L. Humphreys, Tiffany G. Woynaroski

**Affiliations:** 1 Department of Hearing & Speech Sciences, Vanderbilt University, Nashville, Tennessee, USA; 2 Frist Center for Autism & Innovation, Vanderbilt University, Nashville, Tennessee, USA; 3 Department of Psychology and Human Development, Vanderbilt University, Nashville, Tennessee, USA; 4 Department of Hearing & Speech Sciences, Vanderbilt University Medical Center, Nashville, Tennessee, USA; 5 Neuroscience Graduate Program, Vanderbilt University, Nashville, Tennessee, USA; 6 Vanderbilt Brain Institute, Vanderbilt University, Nashville, Tennessee, USA; 7 Vanderbilt Kennedy Center, Vanderbilt University Medical Center, Nashville, Tennessee, USA; 8 Department of Communication Sciences and Disorders, John A. Burns School of Medicine, University of Hawaii at Manoa, Honolulu, Hawaii, USA

**Keywords:** autism, caregiving, infant siblings, measurement, parent mental health, stress

## Abstract

This study measured experiences of parenting stress and stressful life events in caregivers of families with a toddler who has either an autistic or non-autistic older sibling(s). Caregivers of toddlers (12–18 months old) with older autistic siblings (Sibs-autism; *n* = 58) and toddlers with older non-autistic siblings (Sibs-NA; *n* = 46) completed questionnaires assessing stress related to parenting their toddler and their exposure to stressful life events since their toddler’s birth. We compared levels of parenting stress and stressful life events between caregivers of Sibs-autism and Sibs-NA and examined the association between these measures. Caregivers of Sibs-autism reported significantly higher levels of parenting stress and stressful life events relative to caregivers of Sibs-NA, with small to moderate effects. Parenting stress and stressful life events were moderately correlated. Across these groups of caregivers, parenting stress and stressful life events appear to be related, but partially distinct aspects of caregiver stress. These findings highlight the importance of assessing multiple aspects of stress to better understand how stress may influence both caregiver wellbeing and the development of children with autistic siblings.

## Lay Summary

In this study, we compared the stress levels of caregivers who care for toddlers with older siblings diagnosed with autism to the stress levels of parents/guardians of toddlers with older siblings who are not diagnosed with autism. We assessed perceived parenting-related stress and obtained reports on the number of stressful life events the parents/guardians had experienced since the toddlers in the study were born. We found that parents/guardians of toddlers with older autistic siblings reported higher levels of parenting stress and more stressful life events relative to parents of toddlers without older autistic siblings. Future work should study how to best support parents/guardians who are experiencing high levels of stress.

## 1 | Introduction

In families with autistic children, or who have a child at increased likelihood for having a neurodevelopmental condition, researchers have called for increased consideration of caregiver factors that may cascade onto child development (Bradshaw et al. 2022; Choi et al. 2020; Derguy et al. 2016; Mailick et al. 2025; Weitlauf et al. 2020; Yoder et al. 2021). Early in life, children’s main social partners are their caregivers, who are key in scaffolding development. Supportive caregiving environments can buffer against other factors that may hinder development, such as adverse experiences, poverty, or stress (e.g., Henry et al. 2020; Jensen et al. 2017; Markfeld et al. 2023; Zhu et al. 2023). Stress can be conceptualized as including two related components: exposure to external environmental stressors (e.g., stressful life events) and the response to stressors (e.g., perceived psychological stress; Harkness and Monroe 2016). Caregiver stress can negatively impact the caregiver, the caregiver–child relationship, the child, and the broader family system (Derguy et al. 2016; Morgan 1988).

### 1.1 | Stress in Caregivers of Autistic Children

Having a child diagnosed with autism can present a range of unique challenges for the family system (Cridland et al. 2014; Morgan 1988). More specifically, caregivers of autistic children may be at increased risk for greater exposure to stressful life events and for elevated perceived stress related to parenting (Ellis et al. 2011). Indeed, researchers have found that caregivers of autistic children experience significantly more stress than caregivers of non-autistic children and caregivers of children with other neurodevelopmental conditions such as Down syndrome (Bitsika et al. 2013; Bitsika and Sharpley 2004; Estes et al. 2009; Hayes and Watson 2013). For example, a meta-analysis by Hayes and Watson (2013) found that caregivers of autistic children reported higher levels of perceived stress related to parenting their autistic child relative to caregivers of all other children. Other studies have found that, compared to caregivers of non-autistic children, caregivers of autistic children are more likely to experience stressful life events such as divorce (Bahri et al. 2023; Hartley et al. 2010; Risdal and Singer 2004), reduced healthcare access due to a minoritized identity (e.g., Mandell et al. 2009), and financial strain due to paying for services for their child(ren) and/or a caregiver leaving a job to care for their child (e.g., Amendah et al. 2011).

### 1.2 | Rationale for Studying Siblings of Autistic Children and Their Caregivers

However, past work investigating caregiver stress in autistic children has been limited by measuring stress relatively late in childhood. To study early influences on child development in families with autistic children, researchers now often follow infant siblings of older autistic children (i.e., Sibs-autism) from infancy through early childhood. Sibs-autism are approximately 8–10 times more likely to be diagnosed with autism compared to infants and toddlers with older, non-autistic siblings (i.e., Sibs-NA; Messinger et al. 2013; Ozonoff et al. 2011, 2018). Additionally, Sibs-autism who do not go on to have autism are also more likely to have language delay and/or disorder and to present with subclinical autism features (Marrus et al. 2018). Given their elevated likelihood of autism and other developmental differences, Sibs-autism represent a key group for understanding early developmental pathways. Studying caregiver stress in these families during toddlerhood can inform understanding of factors in the family system that may interact with neurodevelopmental differences. Additionally, given that caregivers of Sibs-autism often serve as coaches or interventionists during preemptive interventions for their young child (e.g., Hampton and Rodriguez 2022), understanding how stress may differ for caregivers of Sibs-autism as compared to caregivers of Sibs-NA is an important first step in characterizing the broader context of these caregivers. Thus, the present study uses an infant siblings design to explore levels of caregiver stress in a group of caregivers of Sibs-autism and Sibs-NA, with the goal of understanding how these groups of caregivers may differ in their stress levels as they pertain to their young toddler, who is a sibling of either an autistic or non-autistic child.

### 1.3 | Previous Work on Stress in Autistic Siblings Research

Findings of elevated stress among caregivers of autistic children would suggest that caregiver stress is likely to be higher with respect to Sibs-autism than Sibs-NA. Through the lens of family systems theory (Cridland et al. 2014), the adaptation of the Sibs-autism family system to accommodate autism-specific needs for the older sibling(s)—and to support the development of the younger child who is at increased likelihood for autism— may increase parenting stress not only in relation to parenting the autistic child but *also* in parenting Sibs-autism. In line with prior research documenting heightened likelihood of stressful life events in caregivers of autistic children, it stands to reason that caregivers of Sibs-autism may also experience more stressful life events within the lifetime of their Sibs-autism child than caregivers of Sibs-NA (Amendah et al. 2011; Nealy et al. 2012; Risdal and Singer 2004). Nonetheless, the only study to investigate any aspect of caregiver stress in caregivers of Sibs-autism is our prior study (Markfeld et al. 2023). We found that caregivers of Sibs-autism reported greater stress related to parenting their Sibs-autism as indexed by the Parenting Stress Index (PSI; Abidin 2012) than did caregivers of Sibs-NA, with effect sizes in the small to medium range. However, these differences were not statistically significant, likely due to low statistical power given the relatively small sample size. No previous study has examined differences in exposure to stressful life events in caregivers of Sibs-autism compared to caregivers of Sibs-NA during the lifetime of the infant sibling.

### 1.4 | Jointly Investigating Parenting Stress and Exposure to Stressful Life Events

Additionally, examining both stressful life events and perceived parenting stress could inform models of how caregiver stress influences child development in caregivers of Sibs-autism. In the general population, both elevated parenting stress and greater exposure to stressful life events among caregivers have been associated with poorer outcomes for children, including greater emotional and behavioral problems (Crnic et al. 2005; Ribas et al. 2024). Stressful life events may also exacerbate parenting stress, including within families with autistic children (Derguy et al. 2016). Nonetheless, stressful life events and parenting stress are distinct constructs that are only moderately correlated in the general population (Gauthier-Légaré et al. 2022). As not all caregivers with greater exposure to stressful life events will exhibit elevated parenting stress (and vice versa), attending to both dimensions of stress could help to clarify the facets of stress that may impact caregivers of Sibs-autism.

### 1.5 | Purpose

The purpose of this study was to compare levels of parenting stress and stressful life events between caregivers of Sibs-autism toddlers and Sibs-NA toddlers aged 12–18 months. This study examines parenting stress in a larger sample of caregivers of Sibs-autism and Sibs-NA than in our prior work (Markfeld et al. 2023) and is the first to investigate differences in exposure to stressful life events between these two caregiver groups during the lifetime of the younger sibling. Our research questions were as follows:

1. Do caregivers of Sibs-autism toddlers and Sibs-NA toddlers differ in their reported levels of stress related to parenting their toddler?
2. Do caregivers of Sibs-autism and Sibs-NA differ in their reported levels of exposure to stressful life events within their toddler’s lifetime?
3. To what degree are scores of parenting stress and stressful life events correlated across caregivers of Sibs-autism and Sibs-NA?

We hypothesized that caregivers of Sibs-autism would report higher levels of parenting stress and stressful life events relative to caregivers of Sibs-NA. We also hypothesized that scores of parenting stress and stressful life events would be moderately correlated across caregivers of Sibs-autism and Sibs-NA.

## 2 | Materials and Methods

All study procedures were approved by the Vanderbilt University Medical Center Institutional Review Board, and written informed consent was obtained from caregivers prior to their participation in the study. The use of AI Technology was not used in conceptualizing or developing this study, nor was this technology utilized in the analysis, writing, or review processes.

### 2.1 | Participants

All 104 participants in this study were recruited for an NIDCD-funded project, the **S**ensory **P**roject in **I**nfant/Toddler **S**iblings of Children with Autism (Project SPIS; PI Woynaroski) to evaluate early predictors of language in a sample of Sibs-autism (*n* = 58) and Sibs-NA (*n* = 46). We included two cohorts of participants in this study. The first cohort, previously described in Markfeld et al. (2023), was collected between 2017 and 2019 (*n* = 51). The second cohort was collected between 2021 and 2024 (*n* = 53). Thus, the present sample partially overlaps with samples included in prior reports (e.g., Feldman et al. 2024; Markfeld et al. 2023). Participants were recruited via flyers, university and hospital clinics, community settings (i.e., Adventure Science Center), emails, word of mouth, and social media.

To be included in the study, toddlers in both groups had to (a) have a chronological age of 12–18 months (±30 days) at the first timepoint, (b) have at least one older sibling, and (c) live in a monolingual English-speaking home as confirmed via caregiver report. Toddlers classified as Sibs-autism were required to have at least one autistic older sibling, and Sibs-NA were required to have only non-autistic older siblings. Diagnostic status of older siblings in the Sibs-autism group was confirmed by an independent diagnostic assessment including a caregiver interview and a research-reliable administration of the Autism Diagnostic Observation Schedule, second edition (ADOS-2; Lord et al. 2012) or via Vanderbilt medical record review, when available. Non-autistic status of older siblings in the Sibs-NA group was confirmed by a score below the screening threshold on the Social Communication Questionnaire (Rutter et al. 2003). Toddlers in the Sibs-NA group also had no history of developmental concerns and no family history of autism per caregiver report. Exclusion criteria for toddlers in both groups were (a) presence of a known genetic condition, (b) an adverse neurological history, and (c) pre-term birth (gestation < 37 weeks). All participants had at least one older sibling. The number of older siblings that toddlers had across groups ranged from 1 to 6 (Median = 1, Mean = 1.67). The Mullen Scales of Early Learning (MSEL; Mullen 1995) was administered at Time 1 for sample characterization. See Table 1 for a summary of participant characteristics by sibling group and Table S1 for a summary of participant characteristics further broken down by cohort. Cohorts significantly differed on their MSEL Early Learning Composite scores (*W* = 1704, *p* = 0.02, Hedge’s *g* = 0.48), but did not significantly differ on any other summarized demographic characteristics.

### 2.2 | Procedures

Caregivers completed the following measures when toddlers in the study were between 12 and 18 months of age.

#### 2.2.1 | Parenting Stress

Caregivers across cohorts 1 and 2 completed the PSI as a measure of parenting stress (Abidin 2012). The PSI is a 36-item, validated caregiver report measure (Abidin 2012; Haskett et al. 2006) that has been used in past research with caregivers of autistic children (e.g., Bitsika and Sharpley 2004; Estes et al. 2009; Zaidman-Zait et al. 2011) and Sibs-autism (Markfeld et al. 2023). Caregivers were instructed to answer the PSI questions as they pertained to the younger sibling who was participating in the broader study. We derived the Overall raw score, as well as five subscores (i.e., General Distress, Parenting Distress, Rewards Parenting, Child Demandingness, and Difficult Child) previously validated in caregivers of autistic children and used in prior work from our laboratory (Zaidman-Zait et al. 2011). The General Distress subscore indexes broad, non-parenting-specific stressors (e.g., isolation). The Parenting Distress subscore reflects distress associated specifically with the caregiving role (e.g., feeling trapped by caregiving responsibilities). The Rewards Parent subscore assesses child characteristics that are related to positive caregiver–child interactions (e.g., child giggles while playing). The Child Demandingness subscore captures caregiver perceptions that caring for the child may be unexpectedly difficult (e.g., the child is more of a problem than was expected). The Difficult Child subscore indexes child characteristics that may increase caregiver stress (e.g., emotional dysregulation, difficulty with adaptability). Correlations for the five PSI subscores were moderate to strong in the present sample (*r*s ranged from 0.51 to 0.81; Table 2), and the internal reliability for each subscore was good (Cronbach’s α range = 0.81–0.87). The majority of respondents in both the Sibs-NA group (98%) and the Sibs-autism group (97%) were mothers. One father served as the primary caregiver the Sibs-NA group, and two fathers served as the primary caregiver in the Sibs-autism group.

#### 2.2.2 | Exposure to Stressful Life Events

For toddlers in cohort 2 only, caregivers additionally completed a modified version of the Assessment of Parent and Child Adversity (APCA; King et al. 2022), a validated questionnaire designed to assess both a caregiver’s and their child’s lifetime exposure to stressful experiences. This measure was added for our second cohort following its validation and publication. On the original APCA, caregivers respond yes/no to whether they have experienced 40 different types of adversity, along with two items questions asking about exposure to “other” events not specifically listed. The original version of the APCA allows for calculation of several summary scores indexing caregiver and child exposure to adversity. These summary scores have demonstrated convergent and predictive validity (e.g., with alternate measures of caregiver life experiences, caregiver mental health, and child outcomes; King et al. 2022; Lynn et al. 2023). For further detail regarding the APCA, including how the measure was developed and how each question was worded, please see King et al. (2022) and https://osf.io/tgmpz/.

For the purpose of the present analyses, we focused on caregiver adversity since the target child’s birth to present-day (i.e., from birth to when the child’s age was between 12 and 18 months), which has previously demonstrated validity for predicting child emotional and behavioral problems (King et al. 2022). Three items pertaining to the caregivers’ own childhood adversity were not administered, yielding a total of 37 probed events, along with the two “other” events. We derived the raw count of the number of stressful life events endorsed by caregivers for use in analyses. Caregivers filled out the APCA while a senior member of the study team was present to ensure that they had support and to have any questions related to the APCA answered. Across all families in our study, we provided referrals for services that our interdisciplinary team deemed appropriate during feedback sessions and/or in written reports (e.g., psychotherapy, supportive parenting programs).

### 2.3 | Analytic Plan

Analyses were conducted in *R* version 4.4.1 (R Core Team 2022). We first assessed whether the PSI and APCA scores were normally distributed using a Shapiro–Wilk test. The PSI and APCA overall raw scores were not normally distributed, *W* = 0.93, *p* < 0.001 and *W* = 0.69, *p* < 0.001, respectively. Due to the non-normal distribution of our two variables of interest, we used non-parametric tests in subsequent analyses.

#### 2.3.1 | Missing Data

Three participants were either partially or fully missing PSI data (one participant in cohort 1 partially completed this measure; two participants in cohort 2 did not complete this measure). Fifty-seven participants were missing APCA data due to this measure being added to the study approximately halfway through data collection. We imputed missing PSI scores using the *missForest* package in R (Stekhoven and Bühlmann 2012). We did not impute scores for the APCA due to a low percentage of complete data for this survey relative to our overall sample (i.e., over 50% missingness across cohorts; Enders 2011); thus, the sample size for analyses of stressful life events included 26 Sibs-autism and 21 Sibs-NA.

#### 2.3.2 | Primary Analyses

To answer our first and second research questions regarding differences in perceived and objective stress, we conducted Mann–Whitney *U* tests to assess between-group differences in levels of parenting stress and stressful life events between caregivers of Sibs-autism and Sibs-NA. We quantified the magnitude of these effects using Hedges’ *g* (small effect = 0.2, medium effect = 0.5, large effect = 0.8; Taylor and Alanazi 2023). We examined the un-conditional association between parenting stress and stressful life events using Spearman rank-order correlation tests between and within caregivers of Sibs-autism and Sibs-NA. We also explored whether the five subscores of the PSI differed between caregivers of Sibs-autism and Sibs-NA via Mann–Whitney *U* tests. Finally, we compared item-level endorsements on the APCA between Sibs-autism and Sibs-NA. We chose to report odds ratios due to the small cell sizes, wherein an odds ratio greater than one indicates that caregivers of Sibs-autism were more likely to endorse the event than caregivers of Sibs-NA, with higher odds ratios indicating a stronger association between having a child with autism and endorsing that experience (McHugh 2009). Conversely, an odds ratio less than one indicates that caregivers of Sibs-autism were less likely to endorse the event than caregivers of Sibs-NA. As an example, an odds ratio of 1.5 means that the odds of the event happening in one group are 1.5 times the odds of it happening in the comparison group, while an odds ratio of 0.67 means the odds are reduced by approximately one-third.

## 3 | Results

### 3.1 | Group Differences in Parenting Stress

**FIGURE 1 |:**
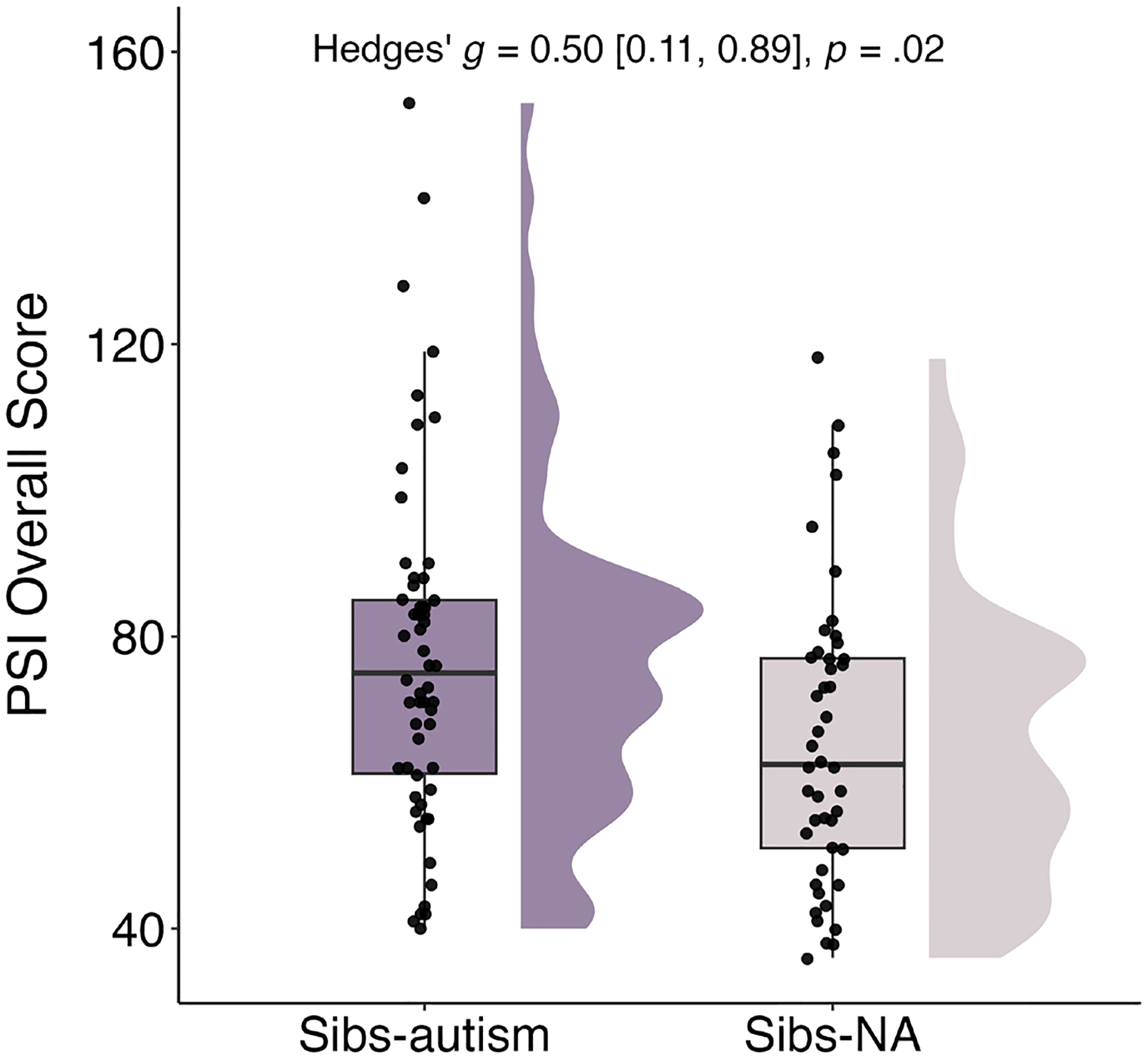
Differences in PSI Overall Raw Scores by Sibling Group. PSI = Parenting Stress Index Short Form, Fourth Edition (Abidin 2012); Sibs-autism = Toddlers with at least one older sibling diagnosed with autism; Sibs-NA = Toddlers with non-autistic older siblings.

Results of the first Mann–Whitney test showed that caregivers of Sibs-autism reported higher levels of parenting stress on the PSI relative to caregivers of Sibs-NA (*W* = 868.5, *p* = 0.02; see Figure 1). This effect was moderate in magnitude (Hedges’ *g* = 0.50; 95% CI [0.11, 0.89]).

### 3.2 | Group Differences in Stressful Life Events

**FIGURE 2 |:**
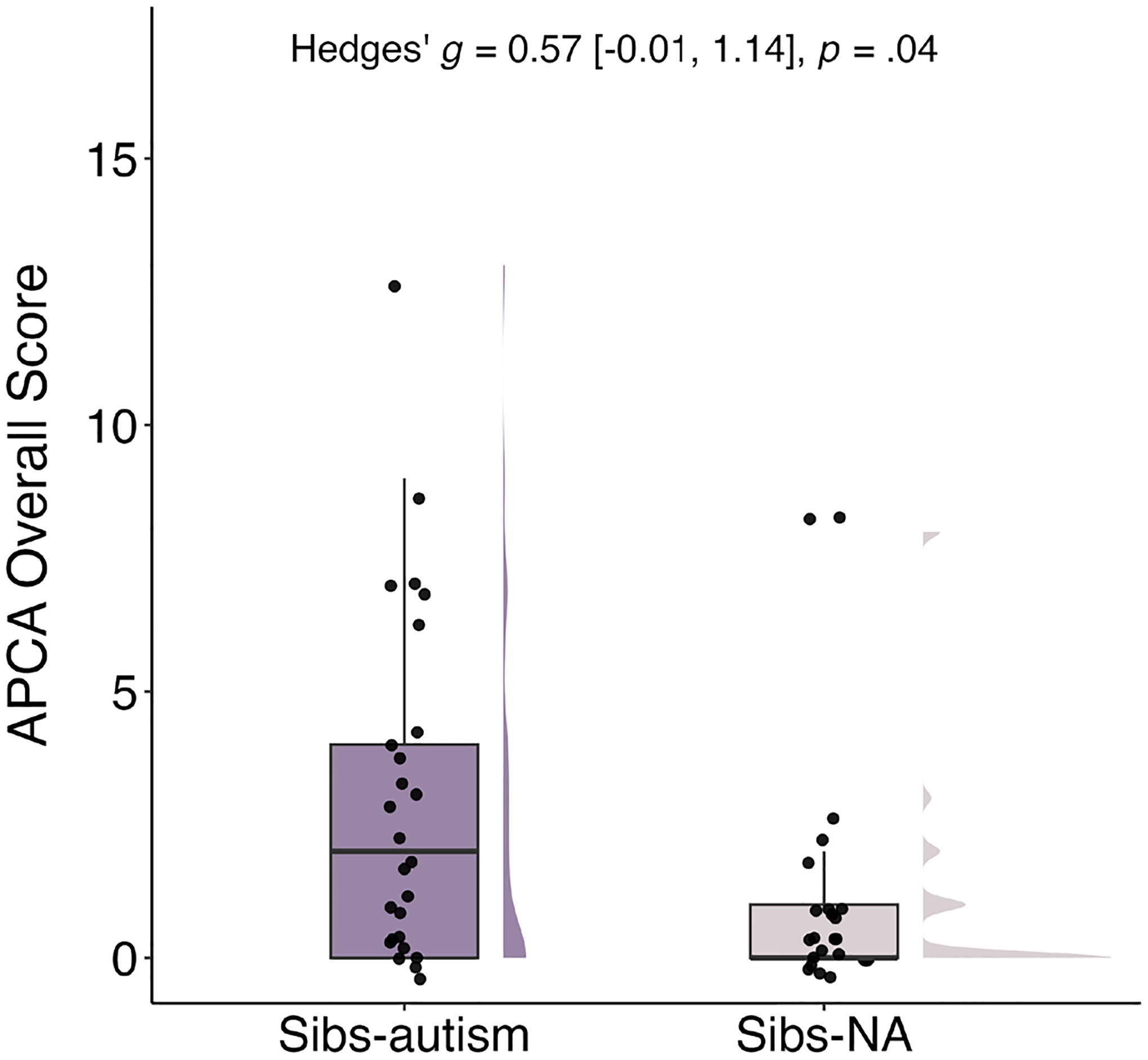
Differences in APCA overall raw scores by sibling group. APCA = Assessment of Parent and Child Adversity (King et al. 2022); Sibs-autism = Toddlers with at least one older sibling diagnosed with autism; Sibs-NA = Toddlers with non-autistic older siblings.

Results of this Mann–Whitney test, in a subset of 47 caregivers (i.e., those from cohort 2), showed that caregivers of Sibs-autism reported experiencing more stressful life events since the birth of the toddler in the study relative to caregivers of Sibs-NA (*W* = 366.0, *p* = 0.04; see Figure 2). This effect was also moderate in magnitude (Hedges’ *g* = 0.57, 95% CI [−0.01, 1.14]).

### 3.3 | Correlations Between Indices of Parenting Stress and Stressful Life Events

**FIGURE 3 |:**
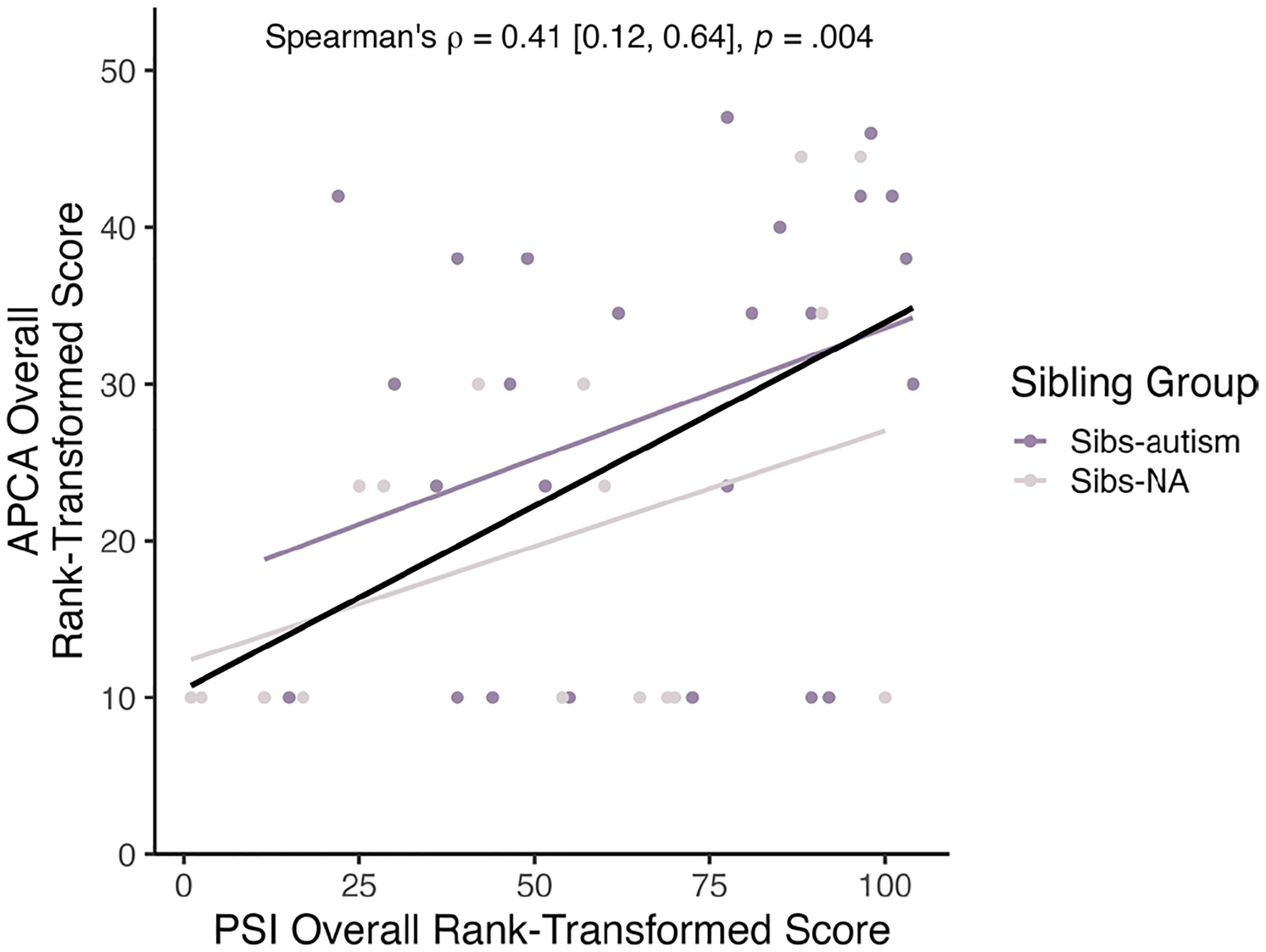
Intercorrelation between parenting stress and stressful life events. PSI = Parenting Stress Index Short Form, Fourth Edition (Abidin 2012); APCA = Assessment of Parent and Child Adversity (King et al. 2022); Sibs-autism = Toddlers with at least one older sibling diagnosed with autism; Sibs-NA = Toddlers with non-autistic older siblings. The black line depicts the relation between PSI and APCA scores across all participants.

We then ran Spearman correlations between PSI and APCA scores to assess the strength of the relation between our indices of parenting stress and stressful life events. PSI and APCA scores were significantly and positively correlated across groups (ρ = 0.41; 95% CI [0.12, 0.64], *p* = 0.004; see Figure 3). This correlation was moderate in magnitude. PSI and APCA scores were also positively correlated within Sibs-autism and Sibs-NA, with similar small-to-moderate magnitudes for Sibs-autism (ρ = 0.37 [−0.03, 0.70], *p* = 0.07) and Sibs-NA (ρ = 0.29 [−0.22, 0.71], *p* = 0.19).

### 3.4 | Post Hoc Analyses

#### 3.4.1 | Group Differences in PSI Subscores

When investigating differences in the PSI subscores (Zaidman-Zait et al. 2011) for caregivers of Sibs-autism versus caregivers of Sibs-NA, we found that groups differed on the subscores of General Distress, Rewards Parent, and Difficult Child, with caregivers of Sibs-autism reporting higher levels of perceived stress in these areas relative to caregivers of Sibs-NA (Table 3). These effects were small to moderate in magnitude (Hedges’ *g* range = 0.44–0.50 across the three subscores with statistically significant group differences). Caregivers of Sibs-autism and Sibs-NA did not significantly differ in their levels of stress as indexed by the Parenting Distress or Child Demandingness subscores (*p* values > 0.11).

#### 3.4.2 | Description of APCA Items Endorsed

Results of exploratory comparisons of item-level endorsements of stressful life events on the APCA between groups are presented in Table 4. Descriptively, caregivers of Sibs-autism endorsed higher frequencies of most types of stressful life events. We observed the largest odds ratios (i.e., greater than 3) for the following items, such that they were more likely to be endorsed by caregivers of Sibs-autism relative to caregivers of Sibs-NA: serving as a caregiver for someone with an illness or disability other than the target child (“for example, a different child, your partner, a best friend, or a parent”), verbal fighting between family members, financial problems, and witnessing the mental illness of someone close other than the target child (“for example, a different child, your partner, a best friend, or a parent”).

## 4 | Discussion

This study is the first to assess stressful life events in caregivers of Sibs-autism and to compare levels of both parenting stress and stressful life events between caregivers of Sibs-autism and Sibs-NA as they pertain to the lifetime of the younger sibling. This study replicates and extends our prior work in a larger sample on parenting stress in caregivers of Sibs-autism and Sibs-NA (Markfeld et al. 2023) and adds to the growing literature focused on stress in families with autistic children as well as families who have a younger child at increased likelihood for autism (e.g., Bitsika et al. 2013; Bitsika and Sharpley 2004; Estes et al. 2009; Hayes and Watson 2013). We found that, on average, caregivers of Sibs-autism reported higher levels of parenting stress and stressful life events relative to caregivers of Sibs-NA, with moderate effect sizes, specifically within their younger child’s lifetime. Groups differed on three of the five PSI subscores derived via guidelines from Zaidman-Zait et al. (2011). Caregivers of Sibs-autism also tended to endorse several specific items on the APCA more frequently than caregivers of Sibs-NA. Finally, we found a moderate association between parenting stress and stressful life events both across and within groups, mirroring prior findings in the general population (Gauthier-Légaré et al. 2022). These findings have important implications for research, theory, and clinical practice.

### 4.1 | Group Differences Within PSI Subscores

Across the subscores of the PSI, caregivers of Sibs-autism reported significantly higher stress levels in the areas of General Distress, Rewards Parent, and Difficult Child compared to caregivers of Sibs-NA (Zaidman-Zait et al. 2011). These subscores may offer additional insights into the unique stressors experienced by caregivers of Sibs-autism. The General Distress subscore includes items such as feeling unable to handle things well and feeling alone and without friends. Higher endorsement of these items among caregivers of Sibs-autism may reflect perceptions of limited resources while caring for at least one child with autism in addition to at least one younger sibling in the present study. The Rewards Parent subscore probes behaviors such as the child rarely doing things that make the caregiver feel good and smiling less than other children. Finally, the Difficult Child subscore reflects child behaviors that caregivers may find challenging, such as the child’s behavior being “more of a problem” than expected. Thus, these subscores may tap broader autism-related features that tend to be elevated in Sibs-autism relative to Sibs-NA, regardless of whether or not the younger sibling eventually receives an autism diagnosis. Differential patterns across subscores of the PSI, as defined by Zaidman-Zait et al. (2011), may inform different clinical recommendations for families.

### 4.2 | Research Implications

Although most prior studies of caregiver stress in autism have relied solely on measures of parenting stress, the present findings suggest that considering both parenting stress *and* stressful life events in future research specifically in caregivers who have a younger child at increased likelihood for autism may be warranted. Item-level responses on the APCA suggest that some stressful life events endorsed more frequently by caregivers of Sibs-autism could relate directly to having an older child with autism (e.g., serving as a caregiver for someone with an illness or disability other than the target child), however, we did not probe whether these events pertained specifically to the older autistic child. Clarifying whether these stressful life events are autism-specific or reflect broader family adversity would help tailor research and clinical interventions to the source of caregivers’ stress. Additionally, the moderate correlation between our measures of parenting stress and stressful life events in the current study suggests that they capture partially distinct aspects of caregiver experience. Considering both aspects of stress among families with autistic children, as well as a younger child at increased likelihood for autism, may improve our ability to identify distinct pathways by which stress affects outcomes and clarify how external stressors and perceived stress interact specifically within Sibs-autism at a relatively young age. In addition, future research might examine the potentially distinct consequences of external stressors versus subjective appraisals of stress. For example, parenting stress—characterized by feelings of being overwhelmed, bothered, or challenged by parenting—may have particularly strong effects on the child, as such perceptions may influence the parent’s relationship with and behavior toward the child (Mak et al. 2020; Nordahl et al. 2020). Future work is needed to investigate the potential generational effects of stress, specifically in Sibs-autism and their caregivers.

### 4.3 | Clinical Implications

Our findings suggest that caregivers of Sibs-autism experience higher levels of both parenting stress and stressful life events as measured during the lifetime of their young child. Given that caregivers of Sibs-autism who participate in early, sometimes preemptive, interventions are often expected to serve as interventionists in the home environment, it is important to consider how caregiver stress may influence intervention efficacy and acceptability (Hampton and Rodriguez 2022). Specifically, elevated exposure to stressful life events among caregivers of Sibs-autism suggests that intervention efforts may benefit not only from addressing child-focused outcomes, but also from incorporating strategies to reduce caregiver exposure to external stressors and mitigating their downstream consequences. Additionally, caregivers reporting higher levels of perceived parenting-related stress could benefit from interventions targeting stress coping and emotion regulation. Providing additional supports and services, such as access to childcare, mental health services, or family support programs, may reduce or mitigate the consequences of caregiver stress in this population, promoting both caregiver wellbeing and more optimal child developmental outcomes. Understanding the stressors that these caregivers may experience can also aid in building clinical rapport and trust between professionals (e.g., speech-language pathologists, psychologists) and the families they serve (Hansen et al. 2024; Miccoli et al. 2022).

Further, our findings add to the growing empirical evidence that caregivers of Sibs-autism may experience unique influences on the family system relative to caregivers of Sibs-NA and suggest that these differences may manifest in elevated levels of parenting stress and stressful life events (Hampton and Rodriguez 2022; Mailick et al. 2025; Markfeld et al. 2023). Caregiver stress may change over time, shifting as toddler siblings’ developmental outcomes unfold, including whether they are later diagnosed with autism or a language delay or disorder. Future work following toddler siblings at increased likelihood for autism longitudinally may benefit from a family systems approach to better understand how caregiver stress interacts with the developing younger sibling, and how these dynamics, in turn, shape broader patterns of family functioning (Cridland et al. 2014; Mailick et al. 2025).

### 4.4 | Strengths, Limitations, and Future Directions

This novel work has multiple strengths. First, to our knowledge, we are the first to measure stressful life events in a sample of caregivers of Sibs-autism and Sibs-NA, providing new insight into the broader range of stressors that caregivers of Sibs-autism may face during the lifetime of their younger child. Second, the Sibs-autism and Sibs-NA groups were matched on several characteristics, including child age and biological sex. Additionally, the diagnostic status of all older siblings was assessed via a research-reliable administration of the ADOS; therefore, we are confident in the internal validity of our between-group comparison (i.e., Sibs-autism versus Sibs-NA).

This study is, however, not without limitations. First, while this study has a larger sample size than our prior work (Markfeld et al. 2023), the sample is still somewhat small—particularly for the subset of caregivers who completed the measure of stressful life events. Future studies should consider collecting measures of parenting-related stress and stressful life events in larger samples of Sibs-autism and Sibs-NA to increase power to detect group differences at the average- and item-level. Further, though the inclusion of a measure of stressful life events in this population addressed an important gap in the literature, the survey that we utilized was still a retrospective, static assessment of exposure to adversity. Such measures may miss the influence of dynamic, day-to-day stressors that shape child development. Future work should consider how static and dynamic measures of both perceived stress and external stressors affect the caregiver–child relationship and influence children’s later developmental outcomes. Further, future work within autism research specifically could also consider comparing and contrasting overall parenting stress with respect to the older autistic child(ren) and the younger child individually. Finally, other family diagnoses, family history, and demographic factors, such as history of psychiatric disorders, childhood adversities for the primary caregivers, and/or caregiver age and occupation, could be considered in future work to better understand how such factors may influence stress levels.

## 5 | Conclusions

The findings of this study advance our knowledge of caregiver stress in Sibs-autism and Sibs-NA. Our results indicate that across indices of parenting stress and stressful life events, caregivers of Sibs-autism report experiencing greater stress relative to caregivers of Sibs-NA as measured with respect to the younger sibling. Future work is needed to investigate how these varying aspects of stress may influence caregiver–child interactions and relate to outcomes of interest in infants at high- and low-familial likelihood for autism and developmental language disorder.

## Supplementary Material

Supplemental Table

Additional supporting information can be found online in the Supporting Information section. **Table S1:** Demographic Information by Sibling Group and Cohort.

## Figures and Tables

**TABLE 1 |:** Participant demographics by sibling group.

	Sibs-autism	Sibs-NA		
	*M* (SD)	*M* (SD)		
	Min–Max	Min–Max	*t*	*p*
Age (months)	15.10 (2.26)	14.51 (2.11)	1.387	0.169
	11–19	11–19		
MSEL Early Learning Composite	93.73 (14.10)	101.04 (9.85)	−3.092	0.002
	70–125	75–121		
Number of older siblings	1.82 (1.24)	1.28 (0.66)	2.871	0.005
	1–6	1–4		
	*n*	*n*	*χ* ^2^	*p*
Biological sex	25 Male	25 Male	0.888	0.346
	33 Female	21 Female		
Race	2 Black	1 Black	1.314	0.723
	52 White	43 White		
	4 Multiple	2 Multiple		
Ethnicity	3 Hispanic or Latino	1 Hispanic or Latino	1.452	0.484
	55 Not Hispanic or Latino	45 Not Hispanic or Latino		
Primary caregiver’s highest level of education	4 12 Years or GED	2 12 Years or GED	8.393	0.136
	15 College/Technical 1–2 Years	6 College/Technical 1–2 Years		
	21 College/Technical 3–4 Years	15 College/Technical 3–4 Years		
	8 Graduate or Professional 1–2 Years	9 Graduate or Professional 1–2 Years		
	6 Graduate or Professional 3–4+ Years	11 Graduate or Professional 3–4+ Years		
	4 Not Reported	3 Not Reported		

*Note:* Early Learning Composite is a commonly used proxy for IQ derived from the MSEL and reported in standard scores (*M* = 100, SD = 15); GED = General Education Development.

Abbreviations: MSEL = Mullen Scales of Early Learning (Mullen 1995); Sibs-autism = Toddlers with at least one older sibling diagnosed with autism; Sibs-NA = Toddlers with non-autistic older siblings.

**TABLE 2 |:** Intercorrelations between PSI factor scores.

Factor	1	2	3	4
1. General distress				
2. Parenting distress	0.65***			
3. Rewards parent	0.62***	0.55***		
4. Child demandingness	0.51***	0.58***	0.74***	
5. Difficult child	0.53***	0.50***	0.72***	0.81***

*Note:* Subscores were derived using guidelines from Zaidman-Zait et al. (2011). Abbreviation: PSI = Parenting Stress Index Short Form, Fourth Edition (Abidin 2012).

*** *p* < 0.001.

**TABLE 3 |:** Comparison of parenting stress by sibling group.

Variable	Sibs-autism (*n* = 58) Median Min-Max	Sibs-NA (*n* = 46) Median Min-Max	*W*	*p*	Hedges’ *g* 95% CI
PSI overall raw score	75.0	62.5	937.5	0.010	0.50 [0.11, 0.90]
	40–153	36–118			
PSI general distress	16.5	15.0	1011.0	0.034	0.44 [0.05, 0.82]
	8–31	8–28			
PSI parenting distress	12.0	10.0	1089.0	0.108	0.31 [0.08, 0.69]
	5–24	5–21			
PSI rewards parent	13.0	10.5	989.0	0.023	0.47 [0.08, 0.86]
	8–28	8–26			
PSI child demandingness	8.0	8.0	1152.5	0.232	0.28 [0.10, 0.67]
	5–25	5–17			
PSI difficult child	15.0	12.5	939.5	0.010	0.50 [0.11, 0.89]
	7–32	7–32			

*Note:* Subscores were derived using guidelines from Zaidman-Zait et al. (2011).

Abbreviations: PSI = Parenting Stress Index Short Form, Fourth Edition (Abidin 2012); Sibs-autism = Toddlers with at least one older sibling diagnosed with autism; Sibs-NA = Toddlers with non-autistic older siblings.

**TABLE 4 |:** Descriptive information and statistics on caregiver exposure to stressful life events.

Type of adversity	*n* (%) Experienced (Sibs-autism)	*n* (%) Experienced (Sibs-NA)	Odds ratio
Abortion/miscarriage	1 (3.7%)	1 (4.8%)	0.76 [0.01, 62.20]
Accident	2 (5.4%)	0	—
Accident (Wit.)	1 (3.7%)	0	—
Arrested/jailed	0	0	0
Authority problems	5 (18.5%)	4 (19.0%)	0.95 [0.18, 5.48]
Caregiver for ill person	6 (22.2%)	1 (4.8%)	5.35 [0.58, 263.90]
Death	7 (25.9%)	4 (19.0%)	1.44 [0.31, 7.74]
Disaster	4 (14.8%)	0	—
Discrimination	2 (5.4%)	0	—
Divorce	5 (18.5%)	0	—
Emotional abuse	3 (11.1%)	0	—
Emotional neglect	4 (14.8%)	0	—
Family arrested/jailed	1 (3.7%)	0	—
Family verbal fighting (Wit.)	6 (22.2%)	1 (4.8%)	5.35 [0.58, 263.90]
Family violence	2 (5.4%)	3 (14.3%)	0.48 [0.04, 4.63]
Financial problems	6 (22.2%)	1 (4.8%)	5.35 [0.58, 263.90]
Fired/laid off	1 (3.7%)	0	—
Immigration	0	0	0
Language barriers	4 (14.8%)	3 (14.3%)	1.03 [0.15, 7.81]
Legal problems	3 (11.1%)	1 (4.8%)	2.41 [0.18, 13.50]
Mental illness (Wit.)	14 (51.9%)	5 (23.8%)	3.08 [0.81, 13.50]
Neighborhood danger	1 (3.7%)	4 (19.0%)	0.17 [0.00, 1.88]
Other exposure	4 (14.8%)	2 (9.5%)	1.60 [0.21, 19.33]
Parental divorce	0	0	0
Partner coercive control	1 (3.7%)	0	—
Partner disagreement	6 (22.2%)	3 (14.3%)	1.65 [0.30, 11.51]
Partner drug abuse	1 (3.7%)	0	—
Partner verbal fighting	6 (22.2%)	3 (14.3%)	1.65 [0.30, 11.51]
Physical abuse	2 (5.4%)	0	—
Physical illness	5 (18.5%)	2 (9.5%)	2.07 [0.30, 23.89]
Physical illness (Wit.)	12 (44.4%)	5 (23.8%)	2.36 [0.61, 10.41]
Police discrimination	0	0	0
Rape	1 (3.7%)	0	—
Robbery, mugging, attack	0	0	0
Robbery, mugging, attack (Wit.)	0	1 (4.8%)	0
Separation from child	0	0	0
Sexual harassment	0	0	0
Sexual molestation	1 (3.7%)	0	—
Someone else exposed	1 (3.7%)	5 (23.8%)	0.13 [0.00, 1.28]

*Note:* Wit. indicates that the caregiver witnessed the adversity. All questions were asked about exposure to the adversity type since the child in the study was born. “Other exposure” refers to the open-ended question, “Have you experienced any other frightening or stressful events that we did not include?” “Someone else exposed” refers to the open-ended question, “Have any of the events mentioned happened to someone close to you so that even though you didn’t see it yourself, you were seriously upset by it?”

## Data Availability

The data that support the findings of this study are available from the corresponding author upon reasonable request.
