# Supplementary material for: Parenting Stress and Stressful Life Events Among Caregivers of Toddler Siblings of Autistic and Non-Autistic Children
Source: Autism Res. Author manuscript; Available in PMC 2026 Apr 17. (PMC13084760; doi:10.1002/aur.70217)
Supplement: Supplemental Table [file NIHMS2162435-supplement-Supplemental_Table.docx]

SI Table 1

|  | Sibs-autism | | Sibs-NA | |  |
| --- | --- | --- | --- | --- | --- |
|  | Cohort 1  (PSI only) | Cohort 2  (PSI + APCA) | Cohort 1  (PSI only) | Cohort 2  (PSI + APCA) |  |
| *n* | 28 | 30 | 23 | 23 |  |
|  | *M* (*SD*)  Min-Max | *M* (*SD*)  Min-Max | *M* (*SD*)  Min-Max | *M* (*SD*)  Min-Max | *p* |
| Age (months) | 14.37 (1.96)  11-19 | 15.80 (2.34)  11-19 | 14.62 (2.20)  11-19 | 14.41 (2.06)  11-18 | .13 |
| MSEL Early Learning Composite | 89.56 (13.28)  70-118 | 97.62 (13.93)  71-125 | 99.04 (9.43)  75-121 | 103.04 (10.07)  85-119 | .02 |
|  | *n* | *n* | *n* | *n* |  |
| Biological Sex | 15 Male  13 Female | 10 Male  20 Female | 12 Male  11 Female | 13 Male  10 Female | .33 |
| Race | 28 White | 2 Black  24 White  4 Multiple | 1 Black  20 White  2 Multiple | 23 White | 1.00 |
| Ethnicity | 1 Hispanic or Latino  27 Not Hispanic or Latino | 2 Hispanic or Latino  28 Not Hispanic or Latino | 1 Hispanic or Latino  22 Not Hispanic or Latino | 23 Not Hispanic or Latino | .70 |
| Primary Caregiver’s Highest Level of Education | 2 12 Years or GED  10 College/Technical 1-2 Years  8 College/Technical 3-4 Years  5 Graduate or Professional 1-2 Years  3 Graduate or Professional 3-4+ Years | 2 12 Years or GED  5 College/Technical 1-2 Years  13 College/Technical 3-4 Years  3 Graduate or Professional 1-2 Years  3 Graduate or Professional 3-4+ Years  4 Not Reported | 4 College/Technical 1-2 Years  7 College/Technical 3-4 Years  5 Graduate or Professional 1-2 Years  7 Graduate or Professional 3-4+ Years | 2 12 Years or GED  2 College/Technical 1-2 Years  8 College/Technical 3-4 Years  4 Graduate or Professional 1-2 Years  4 Graduate or Professional 3-4+ Years  3 Not Reported | .82 |

*Demographic Information by Sibling Group and Cohort*

*Note*. Sibs-autism = Toddlers with at least one older sibling diagnosed with autism; Sibs-NA = Toddlers with non-autistic older siblings; MSEL = Mullen Scales of Early Learning (Mullen, 1995); Early Learning Composite is a commonly used proxy for IQ derived from the MSEL and reported in standard scores (*M* = 100, SD = 15); GED = General Education Development. *p* values depict differences in each variable between Cohort 1 and Cohort 2.
